# The results of meniscal allograft transplantation surgery: what is success?

**DOI:** 10.1186/s12891-020-3165-0

**Published:** 2020-03-12

**Authors:** Henry Searle, Vipin Asopa, Simon Coleman, Ian McDermott

**Affiliations:** 1 University of Edinburgh Medical School, 47 Little France Crescent, Edinburgh, EH16 4TJ Scotland; 2 London Sports Orthopaedics, 31 Old Broad Street, London, EC2N 1HT England; 3 Institute for Sport, Physical Education and Health Sciences, University of Edinburgh, Holyrood Road, Edinburgh, EH8 8AQ Scotland

**Keywords:** Meniscus, Meniscal allograft transplantation, Patient-reported outcome measures

## Abstract

**Background:**

Meniscal allograft transplantation (MAT) may improve symptoms and function, and may limit premature knee degeneration in patients with symptomatic meniscal loss. The aim of this retrospective study was to examine patient outcomes after MAT and to explore the different potential definitions of ‘success’ and ‘failure’.

**Methods:**

Sixty patients who underwent MAT between 2008 and 2014, aged 18–50 were identified. Six validated outcome measures for knee pathologies, patient satisfaction and return to sport were incorporated into a questionnaire. Surgical failure (removal of most/all the graft, revision MAT or conversion to arthroplasty), clinical failure (Lysholm < 65), complication rates (surgical failure plus repeat arthroscopy for secondary allograft tears) and whether patients would have the procedure again were recorded. Statistics analysis included descriptive statistics, with patient-reported outcome measures reported as median and range. A binomial logistic regression was performed to assess factors contributing to failure.

**Results:**

Forty-three patients (72%) responded, mean age 35.6 (±7.5). 72% required concomitant procedures, and 44% had Outerbridge III or IV chondral damage. The complication rate was 21% (9). At mean follow-up of 3.4 (±1.6) years, 9% (4) were surgical failures and 21% (9) were clinical failures. Half of those patients considered a failure stated they would undergo MAT again. In the 74% (32) reporting they would undergo MAT again, median KOOS, IKDC and Lysholm scores were 82.1, 62.1 and 88, compared to 62.2, 48.5 and 64 in patients who said they would not. None of the risk factors significantly contributed to surgical or clinical failure, although female gender and number of concomitant procedures were nearly significant. Following MAT, 40% were dissatisfied with type/level of sport achieved, but only 14% would not consider MAT again.

**Conclusions:**

None of the risk factors examined were linked to surgical or clinical failure. Whilst less favourable outcomes are seen with Outerbridge Grade IV, these patients should not be excluded from potential MAT. Inability to return to sport is not associated with failure since 73% of these patients would undergo MAT again. The disparity between ‘clinical failure’ and ‘surgical failure’ outcomes means these terms may need re-defining using a specific/bespoke MAT scoring system.

## Background

The menisci act primarily as load bearers and secondary stabilisers within the knee joint, and are chondroprotective. Loss of meniscal tissue through trauma or degeneration increases the joint contact forces by up to 350%, exacerbating articular cartilage degeneration [1]. Meniscal tears are particularly common in sportspersons [2, 3] with the typical mechanism of injury involving twisting on a flexed knee or loaded hyperflexion [4]. Symptomatic meniscal tears may require repair, or, if irreparable, a partial meniscectomy [5], significantly increasing the risk of future chondral failure and, ultimately, secondary osteoarthritis [6].

Meniscal Allograft Transplantation (MAT) was first reported in 1989, and is a useful potential option for symptomatic patients where there is loss or discontinuity of the peripheral meniscus and where the degenerative damage is not yet severe enough or the patient is too young for artificial joint replacement surgery to be a suitable option [7–9]. However, MAT is a technically demanding procedure, performed by only a small number of surgeons, and graft availability and cost can be problematic [10]. Patients eligible for MAT are typically young and high-demand, and they are a highly-complex population who have often already endured multiple previous surgical procedures, and many have expectations to return to high activity levels [1]. Although a challenge, restoring function in this patient population is a priority within orthopaedics today [11].

Whilst outcome data is limited by low volumes and short follow-up, there is evidence that MAT can relieve pain, optimising knee function and comfort in selected patients [7]. A pilot randomised-controlled trial in 2018 confirmed MAT had better outcomes at 1 year compared to physiotherapy [12]. Authors have reported positive patient outcome measures following MAT using the KOOS, IKDC, Lysholm, Tegner, and SF-12 scores [13–16]. It remains unclear what proportion of any improvement might be due to MAT alone or from potential concomitant procedures [17], and it is also unclear whether MAT has any long-term chondroprotective effect [18]. Whilst many studies have found that the addition of concomitant procedures at the same time as MAT do not affect outcomes, a recent study showed that compared to isolated MAT, PROMs were improved by the addition of a high tibial osteotomy to offload the affected compartment [19]. Certainly, MAT has been shown to have a higher-failure rate in ACL-deficient knees [20].

Surgical failure is defined variably in studies, but is most often defined as the patient either requiring removal of the allograft, allograft revision or conversion to joint replacement (arthroplasty) [13]. Clinical failure is often defined as a Lysholm Score of < 65 and has been shown not to correlate well with surgical failure or poor patient-reported outcomes [13].

There are differing definitions of failure within the existing literature, with surgical failure not necessarily correlating to clinical failure. The aim of this study was to examine the outcomes of MAT in one surgeon’s patient cohort (using the same graft preparation and fixation techniques), using a variety of surgical and patient-reported outcome measures, with a view to exploring the question of what ‘success’ might actually mean. As with most studies of MAT, many patients required additional concomitant procedures alongside MAT, complicating the assessment of outcomes. It was hypothesized that patients classified as ‘surgical failures’ or ‘clinical failures’ would have significantly lower outcome measures compared to those who were not.

## Methods

Ethical and Risk Assessment Approval was obtained from the University of Edinburgh’s Moray House School of Education, and use of the surgeon’s data was approved by The London Bridge Hospital Clinical Governance Committee, and information governance was maintained throughout the study.

Following informed consent, a retrospective analysis of patients aged 18 to 50 years who had undergone MAT by a single surgeon between 2008 and 2014 was undertaken. The indications for MAT were symptomatic patients with complete or subtotal loss of a meniscus causing pain and reduced function, with evidence of post-meniscectomy degenerative damage where artificial joint replacement surgery was not yet indicated. Each patient had previously been assessed for suitability for MAT by history, clinical examination, imaging and arthroscopic findings, with the principles of treatment fully explained. Alignment and stability were assessed before MAT, to check whether concomitant procedures were also required. Table 1 shows the list of the 31 concomitant procedures performed, with anterior cruciate ligament (ACL) reconstruction being the most common.

**Table 1:** List of Concomitant Procedures

Concomitant Procedure	Number
+ Microfracture	4
+ Chondrotissue cartilage grafting	5
+ ACL reconstruction	14
+ Microfracture	1
+ Chondrotissue cartilage grafting	2
+ PCL reconstruction + Chondrotissue cartilage grafting	1
+ Tibial realignment osteotomy	2
+ Chondrotissue cartilage grafting	2

Sixty patients were identified and sent questionnaires, with zero excluded.

### Surgical procedure

All grafts were fresh-frozen, non-irradiated BioCleansed® menisici obtained from RTI Surgical, Inc., Florida. The grafts were size-matched using the Pollard technique [21, 22] and fixed arthroscopically using transosseuous tibial bone tunnel suture fixation for the anterior and posterior insertional ligaments, plus peripheral capsular sutures, using the method described by Matava et al. [23]. This is considered by many to be the most practical way to secure fixation of the graft [24].

### Rehabilitation

Following surgery, patients were advised to use crutches and a hinged knee brace locked at 0° to 90° for the first 6 weeks. Rehabilitation was then commenced to regain full range-of-motion, with proprioceptive reflex re-training, with slow and cautious muscle strengthening exercises, progressing to light non-impact cardiovascular fitness exercises. Patients were specifically advised to avoid loaded deep knee flexion, heavy weights, loaded twisting or impact through the knee. Patients were specifically advised against returning to any impact type exercise/sport [11].

### Clinical assessment and questionnaire

A request to complete an online self-administered questionnaire was sent via email in December 2016 using QuestionPro software. The date of follow-up was the date the patient completed the online questionnaire. Validated patient-reported outcome measures (PROMs) for knee pathologies (IKDC, KOOS, Tegner and Lysholm) were incorporated into the questionnaire [25]. A Visual Analogue Score (VAS) pain score (0 to 10) and SF-12 Short-Form Health Survey were also used. The SF-12 was also split in the ‘mental’ and ‘physical’ subscales, which were analysed separately. Where questions from the IKDC and KOOS asked about specific joint-loading activities that the patients had specifically been advised not to perform, a ‘Not Applicable’ option was added. Questions on sport were included in the questionnaire, despite the surgeon having specifically advised all patients against ever returning to impact or twisting type activities. Patients were also asked whether they would have the procedure again.

Surgical failure was gathered from clinical documentation, and was defined as removal of most or all of the allograft, allograft revision or conversion to joint arthroplasty. ‘Clinical failure’ was defined as a Lysholm score of < 65. Time from MAT surgery to surgical failure was noted. Time to clinical failure was defined as being from the date of MAT surgery to the date of the patient completing the questionnaire. As there was no continual follow-up, it is acknowledged that clinical failure may have occurred prior to the study’s follow-up. The complication rate for MAT surgery was defined as including those patients who underwent repeat arthroscopy for secondary allograft tears as well as those patients who were surgical failures.

### Statistical analysis

The results were analysed, with descriptive statistics and with PROMs reported as median and range.

Comparisons between PROMs from patients with versus without surgical failure, and with versus without clinical failure were carried out. Data were checked for normality using Shapiro-Wilk tests (Field [26]) with α-level set at 0.05, and if data were normally distributed then independent *t*-tests (α =0.05) were carried out. If data were non-normal, Mann-Whitney U non-parametric tests were performed. Effect sizes were also calculated and classified according to Cohen [27] [30]; small (0.2), medium (0.5), large (0.8). PROMS were also compared between patient groups on whether or not they would have the procedure again (Yes / No / Not sure) using One-Way ANOVA (α =0.05) or Kruskal-Wallis tests, depending on whether data were normal or not. Effect sizes were assessed with Partial Eta Squared with small, medium and large being 0.01, 0.06, 0.14 respectively [26]. The difference between pre- and post-operative Tegner scores was analysed using a repeated measures *t*-test if the data were normally distributed, and a Wilcoxon matched Pairs Signed Ranks test if they were not.

Relationships between five previously studied risk factors (age, gender, number of concomitant procedures, Outerbridge grade, lateral vs medial side of MAT) and surgical and clinical failures were examined with binary multiple logistic regression forced entry method (BMLR) with α =0.05 [26].

## Results

Of the total of 60 MAT procedures performed on 60 patients by this surgeon during the period 2008–2014, 43 (72%) patients responded, with a mean follow-up of 3.4 (±1.6) years. Seventeen patients (28%) did not respond. Table 2 outlines the baseline demographics of the patients.

**Table 2:** Baseline Demographics

Characteristic	Total
Number of Meniscal Allograft Transplants	43
Lateral/Medial	16/27
Male/Female	31/12
Mean Age in years (±SD)	35.6 (±7.5)
Number of concomitant procedures	
0	12
1	21
2	7
3	3
Outerbridge cartilage grade	
0	2
1	3
2	21
3	1
4	16
Mean time to follow up in years (±SD)	3.4 (±1.7)

Data were not normally distributed for all the PROMs except the IKDC and VAS scores. Therefore Table 3 shows the median and interquartile PROM scores for the whole cohort and for the surgical failure and clinical failure groups. For 3 patients, their responses to the IKDC were < 90% complete, rendering them invalid, and these were therefore excluded. All KOOS scores were valid.

**Table 3:** Median (Interquartile Range) Patient-Reported Outcome Measures (PROMs) for whole sample, surgical and clinical failure groups

PROM	Sample (*n* = 43)	Surgical Failure Yes (*n* = 4)	Surgical Failure No (*n* = 39)	Clinical Failure Yes (*n* = 9)	Clinical Failure No (*n* = 34)
KOOS	80.0 (24.4)	66.6 (38.1)	81.0 (24.3)	58.3 (19.4)	82.1 (14.1) ***
IKDC ^a^	55.8 (29.3)	46.6 (37.1)	59.8 (29.3)	43.0 (17.3)	64.4 (26.5) **
VAS	3.0 (3.0)	4.5 (3.0)	3.0 (2.0)	5.0 (3.0)	3.0 (2.0) **
Tegner pre-operative	7.0 (3.0)	7.0 (4.0)	7.0 (3.0)	6.0 (3.0)	7.0 (3.0)
Tegner post-operative	4.0 (3.0)	2.5 (4.0)	4.0 (3.0)	2.0 (2.0)	5.0 (2.0) **
SF-12	40.0 (7.0)	39.5 (19.0)	40.0 (7.0)	35.0 (16.0)	40.0 (7.0) *
SF-12 Mental	22.0 (4.0)	21.5 (10)	22.0 (4.0)	21.0 (10.0)	22.0 (4.0)
SF-12 Physical	17.0 (4.0)	17.0 (4.0)	18.0 (9.0)	14.0 (5.0)	18.0 (4.0) *
Lysholm	81.0 (25.0)	59.5 (54.0)	81.0 (23.0)	51.0 (26.0)	88.0 (18.0) ***

^a^ IKDC responses incomplete for three patients so sample numbers reduced to (40,4,36,9,31).

* - significant at *p* < 0.05

** - significant at *p* < 0.01

*** - significant at p < 0.001

The complication rate was 21%. Four patients were classified as surgical failures and nine were classed as clinical failures, with three patients having both surgical and clinical failure. The mean times to surgical and clinical failures were 1.8 and 2.6 years respectively. Statistical comparison tests did not show any significant differences for any of the PROMs between the two groups. However, Effect Sizes for KOOS and Lysholm scores were large. The Effect Sizes for IKDC, post-operative Tegner scores and SF-12 mental subscale were medium-large and SF-12 (whole test) was small-medium. There was no effect between the two surgical failure groups for pre-operative Tegner Activity Scale. For clinical failure, there were significant differences (*p* < 0.001) between groups for KOOS and Lysholm scores, for the IKDC, the VAS and the post-operative Tegner values (all *p* = 0.002), for the SF-12 (*p* = 0.041) and SF-12 physical subscale (*p* = 0.024) but not for pre-operative Tegner. Effect Sizes for all PROMs were large, except for pre-operative Tegner score, which was small-medium. The Wilcoxon test showed that there was a significant difference (*p* < 0.001) between pre- and post-operative Tegner scores for the whole sample, and this also had a large effect size.

Six patients stated that they would not have the procedure again, with two of these being clinical failures, one being a surgical failure and clinical failure, but three of these did not come under either of the definite failure categories. Five patients were unsure whether they would repeat the procedure again, with the remaining 32 stating they would undergo it again. Table 4 shows the PROM scores grouped by patients’ statements about whether or not they would undergo the procedure again (Yes / No / Not Sure). There were no statistical differences between the groups, although the KOOS (*p* = 0.091), SF-12 physical subscale (*p* = 0.085) and Lysholm scores (*p* = 0.056) approached significance. Effect sizes between the groups were large for KOOS and Lysholm values, medium-large for the SF-12 physical subscale and small-medium for all other PROMs.

**Table 4:** Median (Interquartile Range) Patient-Reported Outcome Measures (PROMs) against whether patients would have the procedure again

PROM	Yes (*n* = 32)	No (*n* = 6)	Not Sure (*n* = 5)
KOOS	82.1 (21.4)	62.2 (39.5)	79.8 (20.2)
IKDC ^a^	62.1 (28.2)	48.5 (40.8)	49.4 (19.6)
VAS	3.0 (2.0)	4.5 (4.0)	3.0 (3.0)
Tegner pre-operative	7.0 (3.0)	6.0 (1.0)	6.0 (4.0)
Tegner post-operative	4.0 (3.0)	3.5 (3.0)	2.0 (4.0)
SF-12	40.0 (8.0)	38.5 (18.0)	37.0 (6.0)
SF-12 Mental	22.0 (4.0)	22.0 (12.0)	23.0 (4.0)
SF-12 Physical	18.0 (4.0)	16.0 (6.0)	15.0 (3.0)
Lysholm	88.0 (25.0)	64.0 (48.0)	73.0 (16.0)

^a^ IKDC responses incomplete for three patients so sample numbers reduced to (29,6,5).

Full results for the BMLRs are shown in Tables 5 and 6. The regression found none of the five risk factors was significantly related to the chance of surgical failure, although gender (being female) was nearly significant (*p* = 0.064). In the regression for clinical failure, again, none of the risk factors was significant, although gender (*p* = 0.077) and number of concomitant procedures (*p* = 0.067) were nearly significantly linked to the chance of clinical failure.

**Table 5:** Binomial logistic regression for surgical failure

Variable	b	Standard Error	Wald	*p*-value	Exp(B)
Age	0.022	0.078	0.081	0.776	1.023
Gender	2.506	1.352	3.434	0.064	12.260
No. of concomitant procedures	0.596	0.927	0.414	0.520	1.815
Outerbridge grade	0.167	0.730	0.053	0.819	1.182
Lateral vs medial	−0.064	1.551	0.002	0.967	0.938
Constant	−5.449	3.618	2.269	0.132	0.004

**Table 6:** Binomial logistic regression for clinical failure

Variable	b	Standard Error	Wald	p-value	Exp(B)
Age	0.076	0.065	1.365	0.243	1.079
Gender	1.843	1.042	3.130	0.077	6.314
No. of concomitant procedures	1.299	0.709	3.355	0.067	3.665
Outerbridge grade	0.131	0.490	0.072	0.789	1.140
Lateral vs medial	1.056	1.069	0.976	0.323	2.876
Constant	−7.144	3.269	4.776	0.029	0.001

Pre-operative and post-operative patient sporting activity, using a combination of findings from experimental studies and clinical investigations to define intensity of impact [28], is shown in Table 7. Thirty two (74%) patients returned to fewer, lower intensity sporting activities following MAT, with 34 patients (79%) expecting this change. Of those who did not return to the same level of sport, 73% stated that they would undergo the procedure again. At review, 39.2% were satisfied, 20.9% were neither satisfied nor dissatisfied, and 40% were dissatisfied with their level of sporting activity achieved following surgery.

**Table 7:** Sporting activities undertaken before and after MAT

Sports Category	Sport	Percentage playing before MAT	Percentage playing after MAT	Category of joint loading
Ball Sports	Soccer	8%		High
	Rugby	7%		High
	Golf	6%	10%	Low
	Cricket	5%		Medium
	Field Hockey	2%		High
	Netball	2%		High
	Lacrosse	1%		High
	Basketball	1%		High
	Volleyball	1%		High
	Ultimate Frisbee	1%		High
Racquet Sports	Tennis	7%	4%	High
	Squash	3%		High
	Badminton	2%		High
Adventure Sports	Rock Climbing	2%		Medium
	Paragliding	1%		Medium
	Motocross	1%		High
Combat Sports	Tae-kwon do	1%		High
	Boxing	1%	2%	Medium
Snowsports	Skiing	11%	4%	Low
Aerobic Sports	Running	11%		High
	Walking	5%	17%	Low
	Roller-blading		2%	High
Watersports	Swimming	6%	15%	Low
	Waterskiing	6%		High
Cycling	Road Cycling	12%	42%	Medium

## Discussion

This study sought to record the PROMs of patients who had undergone MAT, and to investigate whether there were differences reported in those patients with ‘surgical failure’ or ‘clinical failure’ versus those categorised as being ‘successful’.

For the whole sample, PROM scores showed generally good outcomes, with median KOOS, IKDC and Lysholm scores being 80, 55.8 and 81. Further investigation showed that there was a trend towards PROMs being lower in those patients with surgical failure, although this was not statistically significant. This was possibly due to the small sample of surgical failures (*n* = 4). Patients classified as clinical failures did have significantly lower KOOS and IKDC scores, and lower post-operative Tegner values. In the present study, 74% of patients said that they would undergo MAT again. The median post-operative Lysholm, KOOS and IKDC scores in this sub-group were 88, 82, and 62.1 - scores consistent with good outcomes following MAT. However, there were no significant differences in PROMs between those who stated that they would undergo the procedure again compared to those who stated that they would not and those who were not sure.

The results for Lysholm, KOOS and Tegner scores compared favourably or similarly to other reported series in the published literature [14–16], although our IKDC scores were lower [15]. Naimark et al. reported similar outcomes in patients who underwent arthroscopic partial meniscectomy [29]. The Tegner scores were significantly reduced following surgery, probably due to the specific post-operative restrictions that were recommended by the operating surgeon.

It should be noted that there are other techniques to replace menisci, such as the Actifit and the Collagen Meniscal Implant. Interestingly, the studies evaluating the effect of these treatments also do not have a consensus on accepted definitions of failures [30]. However, these implants are only suitable specifically for the treatment of partial meniscal loss where the is still continuity of the peripheral meniscal rim, as they require a meniscal rim for attachment [31]. This therefore makes comparison to MAT difficult. De Coninck et al. reported more favourable outcomes in IKDC with the Actifit compared to our study, but our study showed higher results in KOOS and Lysholm scores [32]. The two studies had comparable mean ages, but De Coninck excluded patients who had severe cartilage damage, whilst in our study 44% had an Outerbridge Grade III or IV, and this may explain the higher IKDC scores in their study. Furthermore, the KOOS and IKDC scores both contain questions regarding activities such as jumping, twisting and pivoting; activities which our participants were advised not to undertake, and so may bias results, as other studies also report higher scores in these questionnaires [33, 34].

While the number of patients undergoing MAT remains small, finding the right tool to evaluate patients is problematic. New scoring systems may provide improved evaluation of MAT.

The Western Ontario Meniscal Evaluation Tool (WOMET) may be appropriate. It is a specific questionnaire aimed to detect the impact of meniscal tears on health-related quality of life [35]. When first designing this present study, it was decided not to include WOMET as there were doubts at that time with regard to WOMET’s reliability, construct validity and responsiveness [36]. It would also have caused questionnaire fatigue by adding a raft of further questions which may have reduced questionnaire completion rates. Whilst Sgroi et al. reported superior measurement of knee function and quality-of-life impairment using WOMET compared to the KOOS in patients treated for meniscal tears [37], to the authors’ knowledge, it had not been used in studies purely investigating MAT. Whether WOMET is indeed a more useful tool for the evaluation of patients who have undergone MAT, however, is a topic in itself for further research. Until the most appropriate and validated scoring system is established for the evaluation of patients who have undergone MAT, this present study suggests that success cannot be adequately defined.

The surgical failure rate of 9% was similar to that reported in other comparable studies, which have reported a failure rate of 10.4% [13]. The clinical failure rate of 21% was greater than that reported by Lee et al. [16] (8.5%) and Zaffagnini et al. [14], (11%). The complication rate was 21%; similar to the 21.3% rate reported in the meta-analysis by El Attar et al. [7]. A consensus regarding the current definition of clinical and surgical failure is required, since two (50%) patients with surgical failures and six (66.7%) with clinical failures said that they would undergo the MAT procedure again or were unsure. It is concerning that previous authors have solely used these endpoints to report the efficacy of MAT.

This study, therefore, suggests that the term ‘patient satisfaction’ may define success better than ‘surgical failure’ or ‘clinical failure’. Patient satisfaction, in terms of activity levels and current symptoms, do not in this study correlate with surgical outcomes. Patient satisfaction may be best incorporated into a questionnaire using the WOMET score, but again, this requires further research and discussion with specific patient focus groups.

The BMLR analysis did not find any risk factors that were significantly associated with failure, although gender (surgical and clinical failure) and number of concomitant procedures (clinical failure) were nearly significant. However, given the heterogeneity of concomitant procedures, future studies could investigate the effect of these different procedures on the success of MAT. The odds ratios showed that except for lateral (vs medial) MAT in surgical failure, all risk factors increased the odds of surgical or clinical failure. However, many of these odds ratios were close to unity, reinforcing their lack of statistical significance.

This study’s analyses support the findings of Parkinson et al. [13] and Ahn et al. [38] that potential risk factors of age, gender and number of concomitant procedures were not related to surgical failure. However, our study disagrees with those studies, in that Outerbridge classification and lateral vs medial were not found to be significant factors in our analysis. Whilst some studies have found that articular cartilage damage at the time of surgery and medial (vs. lateral) allografts are associated with a higher risk of surgical failure [13], this study supports the findings of other papers that conclude that cartilage damage does not make a difference [38–40] and that medial allografts are not at a higher risk of surgical failure [40]. Importantly, however, it should be noted that where articular cartilage damage was found in the relevant compartment of a patient’s knee at the time of MAT surgery, the articular cartilage pathology was also treated at the same time; with radiofrequency coblation chondroplasty for unstable or rough partial thickness chondral damage, with microfracture for small (<2cm^2^) full-thickness defects or with articular cartilage grafting (using Chondrotissue grafting) for larger (>2cm^2^) defects. Therefore, it could be surmised that appropriate concomitant treatment of articular cartilage lesions possibly lessens or even eliminates the otherwise increased risk of failure after MAT that might otherwise be associated with increasing severity of articular cartilage damage.

One reason for the lack of significance found in some of our analyses might be the small sample size in the present study, combined with the number of risk factors examined, which could have led to a lack of statistical power. The methods in the present study were better controlled compared to some previous research: only fresh-frozen meniscal allografts (which are biologically and mechanically superior to other forms of preservation [41]) were used, and these were all sourced from a single provider, and a single one-surgeon technique was used. Previous authors have either not reported their allograft sources [38, 42] or have used multiple providers with different preservation techniques [13]. Additionally, the generally good outcomes following MAT in the present study may be attributable to the surgical technique; suture fixation of the allograft to the capsule combined with trans-tibial bone tunnel suture fixation is the current preferred technique [14, 43, 44]. Techniques using bone plug fixation risk articular cartilage damage and have been associated with a higher failure rate [45, 46].

There was no difference in the SF-12 mental sub-scores in patients who said that they would undergo surgery again versus those who would not, although the physical sub-score was approaching significance. Saltzman et al. [47] and Rue et al. [48] also previously reported that the physical component improved significantly following MAT but that the mental score did not.

It was observed that following MAT, patients returned to fewer and lower levels of sport. Seventeen patients were satisfied with the level of sport achieved following MAT, but 17 others were dissatisfied. Nearly 80% of patients did expect there to be a difference in the level of physical activity that they could achieve after MAT, although there were no differences between the satisfaction levels between those who expected there to be a difference and those who did not. It is unclear whether the protective post-operative regime or the MAT procedure itself explains the dissatisfaction about not being able to return to sport.

### Limitations

The present study had a number of limitations. First, the sample size was smaller than some comparable studies [13, 38] and so statistical power will have been weakened. However, this was offset by the tight control over materials and methods, and by the use of one surgeon, reducing variability in the procedures. Whilst this ensures homogeneity, longer-term studies are required to ensure a sample size is large enough for an adequately powered analysis for each failure sub-group analysis. MAT is a relatively new procedure in the UK and sample sizes are currently small.

Second, there were no pre-operative PROMs available, apart from the Tegner values. As this was a retrospective study, the collection of pre-operative scores was not feasible. However, this is not unusual, and other authors [13, 38] have also used retrospective methods. As with all retrospective studies, there will be significant recall bias. It is still possible to draw useful conclusions from retrospective studies, but in future more prospective PROMs should be taken prior to MAT surgery.

As there was no continual follow-up, clinical failure may have occurred prior to the time at which the study was performed. It was also not possible in this study to review the potential risk factors of mechanical axis, instability or BMI, as not enough information was available for this within the patient records, and therefore a confounding effect in our results cannot be ruled out. However, in the senior author’s practice it is standard practice to correct any varus or valgus malignment greater than 5^o^ with an appropriate realignment osteotomy either in advance of or at the same time as MAT.

## Conclusions

This retrospective study, with 3-year follow-up of 43 patients undergoing MAT, reported outcome scores that are consistent with success, with scores of 82.1, 62.1 and 88 for KOOS, IKDC and Lysholm, respectively. Those patients classified as clinical failures had significantly lower outcome scores, although the four patients with surgical failure did not, possibly due to the small sample. It was not possible to identify significant pre-operative risk factors associated with surgical or clinical failure, although gender (being female) and the number of concomitant procedures were nearly significant and had high odds ratios. The use of the terms ‘surgical failure’, ‘clinical failure’ and ‘patient satisfaction’ may need further consideration, since up to 60% of patients defined as clinical / surgical failures said that they would undergo the procedure again. Either newer outcome measures, such as the WOMET, or a bespoke MAT-specific measure could help to re-define success / failure and establish a more sensitive marker of graft survival and the anticipated chondroprotective effects of MAT.

## Data Availability

The full datasets generated and/or analysed during the current study are not publicly available due to patient data confidentiality and data protection laws, but an anonymised dataset may be available from the corresponding author upon reasonable request.

## Abbreviations

IKDC: International Knee Documentation Committee
KOOS: Knee Injury and Osteoarthritis Outcome Score
MAT: Meniscal allograft transplantation
SF-12: The 12-Item Short-Form Health Survey
VAS: Visual Analogue Scale
WOMET: Western Ontario Meniscal Evaluation Tool
